# Prediction of Cardiac Arrest in the Emergency Department Based on Machine Learning and Sequential Characteristics: Model Development and Retrospective Clinical Validation Study

**DOI:** 10.2196/15932

**Published:** 2020-08-04

**Authors:** Sungjun Hong, Sungjoo Lee, Jeonghoon Lee, Won Chul Cha, Kyunga Kim

**Affiliations:** 1 Department of Digital Health Samsung Advanced Institute for Health Sciences & Technology Sungkyunkwan University Seoul Republic of Korea; 2 Department of Emergency Medicine Samsung Medical Center Sungkyunkwan University School of Medicine Seoul Republic of Korea; 3 Health Information and Strategy Center Samsung Medical Center Seoul Republic of Korea; 4 Statistics and Data Center Research Institute for Future Medicine Samsung Medical Center Seoul Republic of Korea

**Keywords:** machine learning, cardiac arrest prediction, emergency department, sequential characteristics, clinical validity

## Abstract

**Background:**

The development and application of clinical prediction models using machine learning in clinical decision support systems is attracting increasing attention.

**Objective:**

The aims of this study were to develop a prediction model for cardiac arrest in the emergency department (ED) using machine learning and sequential characteristics and to validate its clinical usefulness.

**Methods:**

This retrospective study was conducted with ED patients at a tertiary academic hospital who suffered cardiac arrest. To resolve the class imbalance problem, sampling was performed using propensity score matching. The data set was chronologically allocated to a development cohort (years 2013 to 2016) and a validation cohort (year 2017). We trained three machine learning algorithms with repeated 10-fold cross-validation.

**Results:**

The main performance parameters were the area under the receiver operating characteristic curve (AUROC) and the area under the precision-recall curve (AUPRC). The random forest algorithm (AUROC 0.97; AUPRC 0.86) outperformed the recurrent neural network (AUROC 0.95; AUPRC 0.82) and the logistic regression algorithm (AUROC 0.92; AUPRC=0.72). The performance of the model was maintained over time, with the AUROC remaining at least 80% across the monitored time points during the 24 hours before event occurrence.

**Conclusions:**

We developed a prediction model of cardiac arrest in the ED using machine learning and sequential characteristics. The model was validated for clinical usefulness by chronological visualization focused on clinical usability.

## Introduction

Clinical decision support systems (CDSSs) analyze data to assist health care providers in making decisions and improving service quality. Recently, artificial intelligence has been widely used in CDSSs, and its importance is increasing [1]. Previous studies have shown that CDSSs that use machine learning are actively applied worldwide and can be very helpful in clinical decision making. CDSSs enable clinicians to consider future possibilities and to develop and implement action plans for patient care. Recently, machine learning techniques have been widely used in various medical fields, including diagnosis or prognosis prediction, pattern recognition, and image classification [2,3].

It is difficult for emergency department (ED) staff to monitor all patients due to limited resources. Thus, precise triage systems that can identify high-risk patients are being considered. For this reason, information technology monitoring systems are important, and the application of machine learning techniques in such systems has been extensively studied [4,5]. These triage systems attempt to predict mortality or cardiac arrest based on patient characteristics. However, few studies of prediction modelling clearly reflect sequential characteristics due to the monitoring process. Moreover, the effectiveness of these systems and their applicability to real-world data have not been adequately investigated. For example, detailed analyses of data processing, imbalance adjustment, and dynamics of various factors are lacking. Accordingly, the clinical impact and usage of prediction models have not been sufficiently investigated. The aims of this study were to develop a prediction model of cardiac arrest in the ED using machine learning and sequential characteristics and to validate its clinical usefulness.

## Methods

### Study Setting

This retrospective study was conducted at Samsung Medical Center, a tertiary academic hospital in South Korea with approximately 2000 beds and an average of 200 ED patients per day. Data were obtained from the electronic medical record hospital database from January 1, 2013 to December 31, 2017. Moreover, data from the National Emergency Department Information System (NEDIS) were collected. NEDIS is a real-time management system for information on patients visiting emergency medical institutions. The NEDIS data contain patient demographics and clinical information, such as age, sex, and clinical outcomes. We followed the guidelines for transparent reporting of a multivariable prediction model for individual prognosis or diagnosis [6]. The study was approved by the institutional review board of Samsung Medical Center (IRB No. 2018-10-025).

### Study Participants

**Figure 1:**
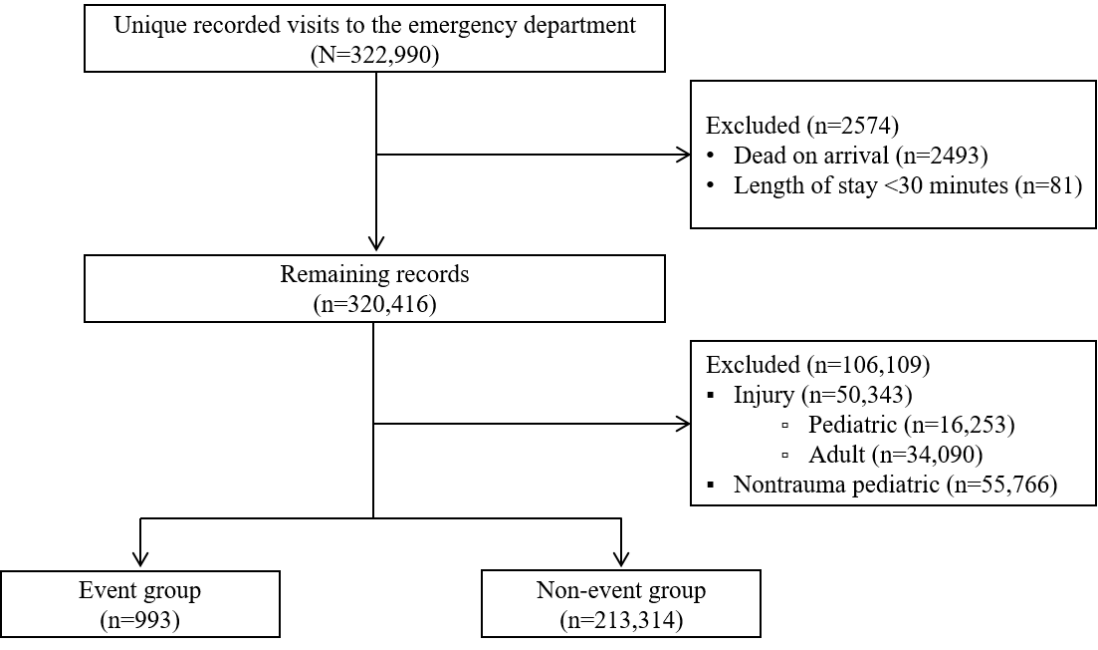
Flow diagram for the selection of the study cohort. Data were processed as unique records based on the date on which a patient visited the emergency department and may correspond to the same patient.

The study population consisted of all ED patients in the study period. The following patients were excluded: those who were dead on arrival, pediatric patients aged <18 years, patients with injury, patients who suffered cardiac arrest or died within 30 min after visiting the ED, and patients who did not experience the outcome event within 30 days of admission. The remaining patients were chronologically divided into the model development cohort (years 2013 to 2016) and the model validation cohort (year 2017). The validation cohort was used to assess the model performance for temporal generalizability. Most patients only visited the ED once (147,303/208,415, 70.68%); fewer patients visited the ED multiple times, with an average of 3.24 visits per patient. Because emergency visits are mostly not scheduled and the reasons for the visits vary [7], each visit is often treated as an independent subject. Thus, we considered each visit as an independent study subject rather than each patient. Patient information was anonymized and deidentified. A flowchart of the study cohort is presented in Figure 1.

### Study Outcome and Predictors

The primary outcome was cardiac arrest regardless of whether cardiopulmonary resuscitation was performed. We also included patients who suffered cardiac arrest after admission to the inpatient ward from the ED. If cardiac arrest occurred several times, we used the first cardiac arrest.

Two groups of predictors were used for the model: initially assessed predictors (sex, age, and chief concerns) and serially assessed predictors (systolic blood pressure, diastolic blood pressure, heart rate, body temperature, respiratory rate, and peripheral oxygen saturation [SpO_2_]). The derived predictor for time (time interval) was the length of the interval (in minutes) between time points [8]. We set the value range for each vital sign as follows: 1 to 300 millimeters of mercury for systolic blood pressure and diastolic blood pressure, 1 to 200 beats per minute for heart rate, 30 to 44 degrees Celsius for body temperature, 1 to 60 breaths per minute for respiratory rate, and 1% to 100% for SpO_2_. The chief concerns were extracted from the NEDIS data and were combined with the raw data. The main symptoms were classified into 39 codes as part of the initial nursing assessment.

**Figure 2:**
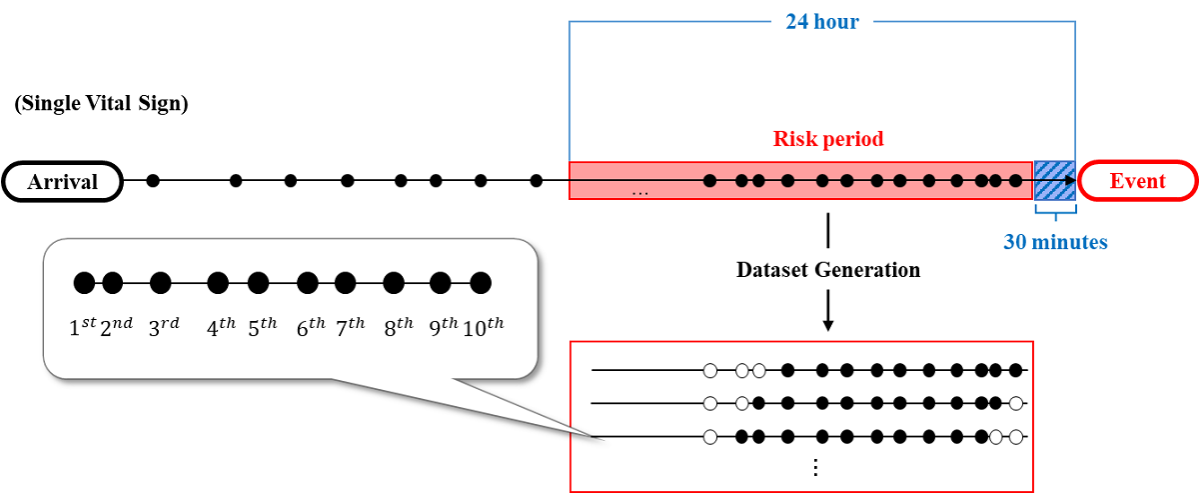
Sequential dataset generation for a single vital sign of one patient. The risk period was defined as a 24-hour interval prior to the event. The 10 consecutive vital signs were grouped as a data set for prediction. Each point represents a single vital sign measurement. This process was applied to other vital signs in the same manner.

The input vector was set to have 10 sequential measurement values for each time point. For example, if a patient’s vital signs were measured 11 times, 11 sets were generated. If the length of the sequential measurements was less than 10, the insufficient data were treated as missing. The 1st and 10th sequence values represent the last and most recent observations, respectively, from the outcome occurrence. We defined the risk period as the interval from 0.5 to 24 hours before outcome occurrence [9]. If the 10th entry of each input vector belonged to the risk period, it was labelled as an event; otherwise, it was labeled as a non-event. These processes are shown in Figure 2.

### Data Preprocessing

Missing data in the sequential measurement values were imputed with the most recent value. If no previous value was available, zero was used [10]. The serially assessed predictors were standardized to have the same range or variability, and the initially assessed predictors were categorized. Our data are affected by the outcome class imbalance problem, which can reduce model performance. To address the imbalance problem, we used undersampling with propensity score matching. Because excessive adjustment may reduce representativeness, we considered various matching ratios, namely 1%, 5%, and 10%, between the event and the non-event groups [11] to determine a suitable ratio. Sex and age were used as matching variables in the propensity score matching based on the R package MatchIt (Multimedia Appendix 1). Data processing was performed using R version 3.4.3 (R Project). Then, statistical analysis was conducted using the Keras and scikit-learn libraries in Python version 3.6.6.

### Analysis

Continuous data are expressed as mean values with the corresponding standard deviations. We performed *t* tests to determine the mean differences between groups. The standardized mean difference is a measure of the effect size for the comparison of two groups [12]. Categorical data were expressed as frequency and percentage. The chi-square test was performed to determine the relationships among categorical features. All tests were two-sided with a statistical significance level of *P*<.05.

To develop a cardiac arrest prediction model, we considered three popular machine learning algorithms, namely logistic regression (LR), random forest (RF), and a recurrent neural network (RNN) [3]. In LR, a ridge penalty was applied to increase the predictive performance and reduce the risk of overfitting [13]. In RF, an entropy criterion was used to measure the split quality [14]. An RNN is an artificial neural network with the advantage of processing sequential data; it is useful for time series analysis using a long short-term memory structure [15]. We used three-layer long short-term memory (the last layer with a sigmoid activation function), an Adam optimization algorithm, and a binary cross-entropy loss function. As a reference cardiac arrest prediction model, we employed the modified early warning score (MEWS) because it is a widely used monitoring tool in ED admission [16]. For optimization, all algorithms used the grid search method. Additionally, the RNN algorithm used the adaptive moment estimation, stochastic gradient descent, and root mean square propagation methods. The hyperparameters in each algorithm were tuned based on 10-fold cross-validation during the model development [17]. To avoid partition bias, the entire cross-validation process was repeated with 5 different partitions. Furthermore, a sensitivity analysis was conducted to assess the effects of the balancing ratio and the influence of the features on the variation of the results among models. More technical details of each algorithm are provided in Multimedia Appendix 2.

To assess the performance of the model, we used various measures, including the area under the receiver operating characteristic curve (AUROC) and the area under the precision recall curve (AUPRC). Also, we used the F1 score to assess class imbalance [18]. Balanced accuracy (BA) was used to determine the optimal cutoff values for the class prediction [19]. Moreover, we used the positive and negative likelihood ratios to assess the clinical usefulness of the prediction model as a diagnostic tool [20]. Calibration and decision curve analyses were conducted to assess the agreement between the observed and predicted values [21,22] and explore the practical threshold for clinical application [23], respectively.

## Results

### Patient Demographics

A total of 322,990 patients visited the ED during the study period. After the exclusion criteria were applied, the final number of patients was 214,307; among these, 993 (0.5%) had the primary outcome of cardiac arrest. We assigned 168,488/214,307 (78.6%) patients to the model development cohort and 45,819/214,307 (21.4%) patients to the model validation cohort. The patient demographics (divided into two groups for each cohort) are shown in Table 1. The number of female patients (114,280/214,307, 52.5%) was greater than the number of male patients. The mean age for the event group was 65.8 years (SD 15.3), whereas the mean age for the non-event group was 55.4 years (SD 17.8).

**Table 1:** Patient characteristics of the development and validation cohorts (N=214,307).

Characteristic			Development cohort				Validation cohort				SMD^a^
			Event (n=791)	Non-event (n=167,697)	*P* value	Event (n=202)		Non-event (n=45,617)	*P* value		
**Demographic data**											
	**Sex, n (%)**				< 001				<.001	0.022	
		Male	472 (59.7)	78,631 (46.9)		133 (65.8)		22,591 (49.5)			
		Female	319 (40.3)	89,066 (53.1)		69 (34.2)		23,026 (50.5)			
	Age (years), mean (SD)		65.2 (15.6)	54.9 (17.8)	<.001	68.3 (13.6)		57.1 (17.6)	<.001	0.119	
**Vital signs, mean (SD)**											
	**Blood pressure (millimeters of mercury)**										
		Systolic	112.6 (25.5)	120.7 (24.1)	<.001	112.9 (28.4)		121.4 (24.8)	<.001	0.033	
		Diastolic	65.0 (15.9)	72.7 (15.0)	<.001	64.3 (16.4)		72.7 (15.0)	<.001	0.002	
	Body temperature (degrees Celsius)		36.7 (2.4)	37.0 (1.7)	<.001	36.8 (2.1)		37.1 (2.1)	<.001	0.053	
	Heart rate (beats per minute)		99.9 (23.7)	88.8 (20.8)	<.001	99.0 (22.1)		88.3 (20.7)	<.001	0.033	
	Respiratory rate (breaths per minute)		21.2 (6.6)	19.8 (3.9)	<.001	20.6 (6.4)		19.1 (3.7)	<.001	0.174	
	SpO_2_^b^ (%)		94.9 (11.0)	90.2 (25.4)	<.001	95.2 (8.7)		96.8 (8.1)	<.001	0.331	

^a^SMD (standardized mean difference) for comparison between the development and validation cohorts.

^b^SpO_2_: peripheral oxygen saturation.

**Figure 3:**
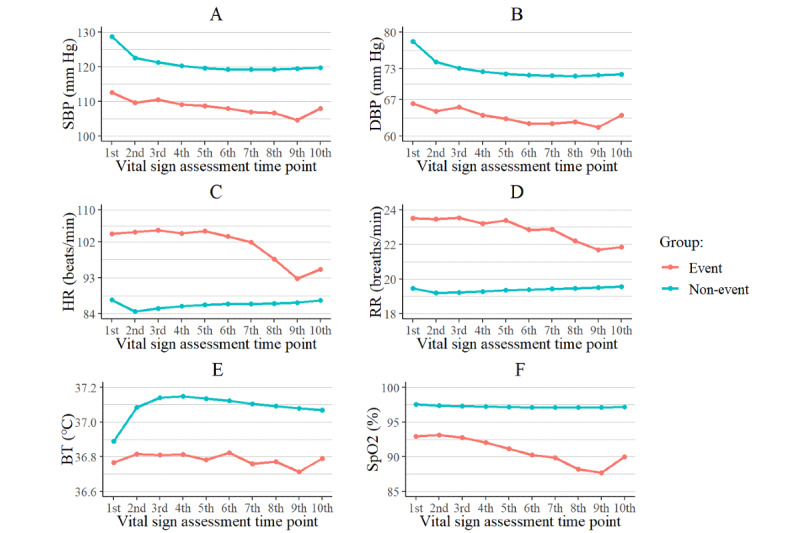
Trends in the event and non-event groups for the vital signs: A. systolic blood pressure; B. diastolic blood pressure; C. heart rate; D. respiratory rate; E. body temperature; F. peripheral oxygen saturation. The x-axis values are the 10 time points before event occurrence, and the y-axis values are the mean values of the vital signs. BT: body temperature; DBP: diastolic blood pressure; HR: heart rate; RR: respiratory rate; SBP: systolic blood pressure; SpO_2_: peripheral oxygen saturation.

Figure 3 shows the average trends of the vital signs for the two groups. Compared to the non-event group, the heart and respiratory rates for the event group were higher on average, whereas the values of the other vital signs were lower. The starting points and the trends were clearly different, demonstrating that the two groups could be distinguished. The chief concern distributions of the groups were different, and dyspnea and abdominal pain were the most common chief concerns in the event and non-event groups, respectively. A comparison of the top 10 chief concerns for each group is shown in Table 2.

**Figure 4:**
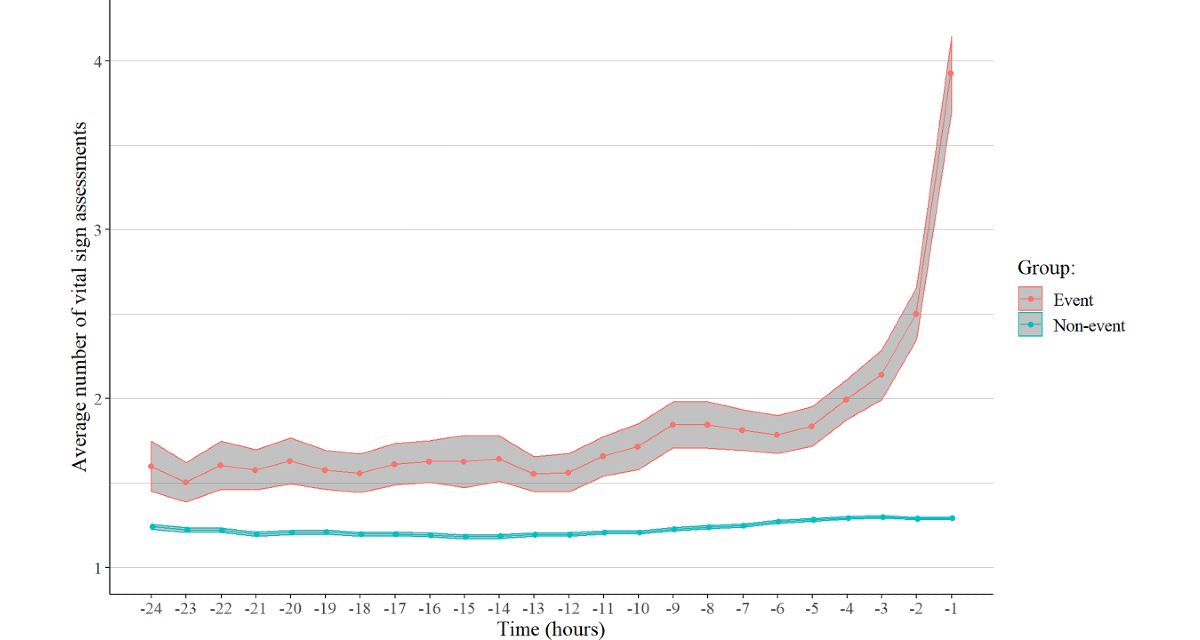
Average numbers of vital sign assessments at each prediction time point for the event and non-event groups. The lines and shaded 95% CIs show the trends for the vital assessments.

**Figure 5:**
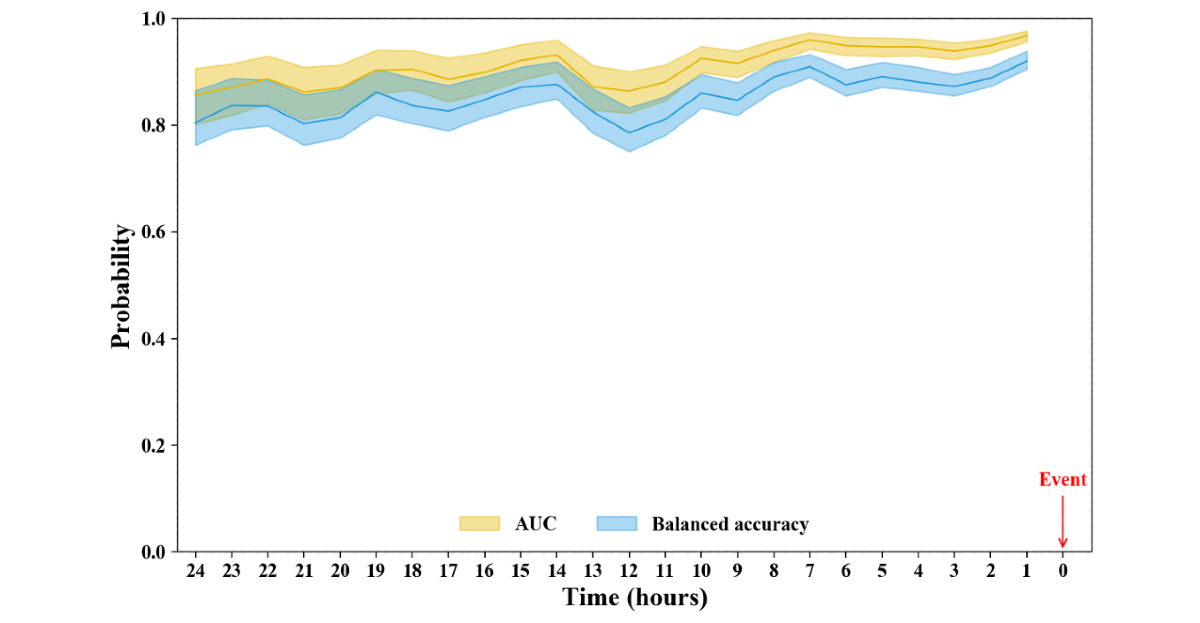
Time point performance in class prediction. The best model was selected based on Table 3, and the predictive performance was evaluated at each prediction time point from event occurrence. The lines and shaded 95% CIs show the trends for the predictive performance. AUC: area under the curve.

Figure 4 shows the frequency difference between the two groups over time and demonstrates that frequent measurements are performed for ED patients in serious condition. Figure 5 shows the model performance over time. It can be seen that the model performance was maintained, with the AUROC remaining at least 80% across the monitored time points during the 24 hours before event occurrence.

**Table 2:** Top 10 chief concerns in the event and non-event groups.

Rank	Event group (n=993)			Non-event group (n=213,314)		
	Chief concern	n (%)		Chief concern	n (%)	
1	Dyspnea	350 (35.25)		Abdominal pain	32 996 (15.47)	
2	Altered mentality	96 (9.67)		Fever	23 551 (11.04)	
3	Fever	88 (8.86)		Dyspnea	16 887 (7.92)	
4	Abdominal pain	64 (6.45)		Dizziness	13,718 (6.43)	
5	Chest pain	60 (6.04)		Headache	9361 (4.39)	
6	General weakness	23 (2.32)		Chest pain	6011 (2.82)	
7	Dizziness	22 (2.22)		Skin rash, urticaria	5042 (2.36)	
8	Chest discomfort	20 (2.01)		Altered mentality	3045 (1.43)	
9	Hematemesis	14 (1.41)		Back pain	2937 (1.38)	
10	Hemoptysis	13 (1.31)		Vomiting	2909 (1.36)	

### Model Performance

Table 3 and Multimedia Appendix 3 summarize the calibrations and overall prediction performance of each model when applying the different balancing ratios for imbalance adjustment, while Table 4 presents the class prediction performance. Model calibrations were described with the integrated calibration index (ICI) and calibration slope. Compared to the other models, the RF model had the smallest ICI in the validation cohort for each balancing ratio (eg, 0.04 for MEWS, 0.04 for LR, 0.02 for RNN, and 0.01 for RF with 10% balancing). The RF-based models showed better calibration in the validation cohort than in the development cohort across the various imbalance adjustments. All the other models showed poorer calibration in the validation cohort than in the development cohort; this suggests that overfitting occurred. As the class imbalance was adjusted with higher balancing ratios, overall improvement was observed for the calibration performance (see the bias-corrected curves in Figure A2 of Multimedia Appendix 3).

**Table 3:** Overall predictive performance for each machine learning algorithm with imbalance adjustment in the development and validation cohorts.

Matching ratio and model			Development cohort							Validation cohort					
			AUROC^a^ (95% CI)		AUPRC^b^ (95% CI)		ICI^c^		Calibration slope (95% CI)		AUROC (95% CI)	AUPRC (95% CI)		ICI	Calibration slope (95% CI)
**0.5% (Real world)**															
	MEWS^d^	0.77 (0.77-0.77)		0.09 (0.08-0.09)		0.013		3.69 (3.64-3.74)		0.80 (0.80-0.81)		0.11 (0.10-0.12)	0.016		4.19 (4.09-4.29)
	LR^e^	0.82 (0.82-0.83)		0.08 (0.08-0.09)		0.003		1.09 (1.08-1.10)		0.82 (0.81-0.83)		0.09 (0.09-0.10)	0.004		1.12 (1.09-1.15)
	RNN^f^	0.96 (0.96-0.97)		0.47 (0.46-0.48)		0.002		1.13 (1.12-1.15)		0.91 (0.90-0.91)		0.17 (0.16-0.18)	0.006		0.70 (0.69-0.72)
	RF^g^	1.00 (1.00-1.00)		1.00 (1.00-1.00)		0.007		6.71 (6.18-7.24)		0.94 (0.94-0.95)		0.37 (0.35-0.39)	0.003		1.09 (1.06-1.13)
**1%**															
	MEWS	0.76 (0.76-0.77)		0.12 (0.12-0.12)		0.022		3.46 (3.41-3.51)		0.79 (0.79-0.80)		0.16 (0.15-0.17)	0.025		4.09 (3.97-4.20)
	LR	0.88 (0.88-0.89)		0.26 (0.25-0.26)		0.008		1.07 (1.06-1.09)		0.88 (0.87-0.88)		0.28 (0.27-0.30)	0.007		1.09 (1.06-1.12)
	RNN	0.96 (0.96-0.96)		0.52 (0.51-0.53)		0.003		1.03 (1.01-1.04)		0.91 (0.91-0.92)		0.33 (0.32-0.35)	0.010		0.79 (0.78-0.81)
	RF	1.00 (1.00-1.00)		1.00 (1.00-1.00)		0.010		7.51 (6.86-8.15)		0.94 (0.93-0.94)		0.47 (0.45-0.49)	0.003		1.14 (1.11-1.18)
**5%**															
	MEWS	0.73 (0.72-0.73)		0.25 (0.25-0.26)		0.052		3.08 (3.01-3.14)		0.77 (0.77-0.78)		0.35 (0.34-0.37)	0.066		4.22 (4.08-4.37)
	LR	0.91 (0.90-0.91)		0.59 (0.58-0.60)		0.034		1.00 (0.99-1.02)		0.90 (0.89-0.90)		0.61 (0.60-0.63)	0.028		1.02 (0.99-1.04)
	RNN	0.97 (0.97-0.97)		0.79 (0.79-0.80)		0.003		1.04 (1.02-1.06)		0.94 (0.93-0.94)		0.68 (0.66-0.69)	0.015		0.82 (0.80-0.84)
	RF	1.00 (1.00-1.00)		1.00 (1.00-1.00)		0.025		9.88 (8.54-11.22)		0.96 (0.96-0.96)		0.78 (0.76-0.79)	0.012		1.21 (1.17-1.25)
**10%**															
	MEWS	0.70 (0.70-0.71)		0.29 (0.29-0.30)		0.018		1.14 (1.11-1.17)		0.76 (0.75-0.77)		0.42 (0.41-0.44)	0.043		1.68 (1.62-1.75)
	LR	0.93 (0.92-0.93)		0.71 (0.70-0.71)		0.043		1.00 (0.99-1.01)		0.92 (0.91-0.92)		0.72 (0.71-0.74)	0.039		0.98 (0.95-1.01)
	RNN	0.98 (0.97-0.98)		0.87 (0.87-0.88)		0.002		1.02 (1.00-1.04)		0.95 (0.95-0.96)		0.82 (0.81-0.83)	0.015		0.81 (0.79-0.84)
	RF	1.00 (1.00-1.00)		1.00 (1.00-1.00)		0.025		10.19 (8.73-11.65)		0.97 (0.97-0.97)		0.86 (0.84-0.87)	0.014		1.14 (1.09-1.18)

^a^AUROC: area under the receiver operating characteristic curve.

^b^AUPRC: area under the precision recall curve.

^c^ICI: integrated calibration index.

^d^MEWS: modified early warning score.

^e^LR: logistic regression.

^f^RNN: recurrent neural network.

^g^RF: random forest.

**Table 4:** Class prediction performance of each machine learning algorithm with imbalance adjustment in the validation cohort.

Matching ratio and model			BA^a^ (95% CI)		Sensitivity (95% CI)		Specificity (95% CI)	F1 score		PLR^b^ (95% CI)		NLR^c^ (95% CI)
**0.5% (Real world)**												
	MEWS^d^	0.75 (0.74-0.76)		0.72 (0.70-0.73)		0.78 (0.78-0.79)		0.096	3.31 (3.24-3.38)		0.36 (0.34-0.38)	
	LR^e^	0.76 (0.76-0.77)		0.75 (0.75-0.78)		0.76 (0.76-0.76)		0.093	3.21 (3.15-3.27)		0.31 (0.29-0.33)	
	RNN^f^	0.84 (0.84-0.85)		0.85 (0.84-0.86)		0.84 (0.83-0.84)		0.143	5.17 (5.09-5.26)		0.18 (0.17-0.19)	
	RF^g^	0.88 (0.88-0.89)		0.88 (0.87-0.89)		0.89 (0.88-0.89)		0.198	7.72 (7.61-7.85)		0.13 (0.12-0.14)	
**1%**												
	MEWS	0.74 (0.73-0.74)		0.72 (0.70-0.73)		0.76 (0.76-0.76)		0.148	2.97 (2.90-3.03)		0.37 (0.36-0.39)	
	LR	0.81 (0.80-0.81)		0.78 (0.76-0.79)		0.84 (0.84-0.84)		0.218	4.77 (4.67-4.88)		0.27 (0.25-0.28)	
	RNN	0.84 (0.83-0.85)		0.87 (0.85-0.88)		0.81 (0.81-0.82)		0.218	4.67 (4.59-4.76)		0.17 (0.15-0.18)	
	RF	0.88 (0.87-0.88)		0.90 (0.89-0.91)		0.86 (0.86-0.86)		0.278	6.49 (6.38-6.60)		0.12 (0.11-0.13)	
**5%**												
	MEWS	0.72 (0.71-0.73)		0.72 (0.70-0.73)		0.72 (0.72-0.73)		0.348	2.57 (2.50-2.63)		0.39 (0.37-0.41)	
	LR	0.85 (0.84-0.85)		0.83 (0.82-0.84)		0.87 (0.86-0.87)		0.555	6.15 (5.97-6.34)		0.20 (0.18-0.21)	
	RNN	0.87 (0.87-0.88)		0.89 (0.88-0.90)		0.85 (0.85-0.85)		0.562	5.96 (5.80-6.15)		0.12 (0.11-0.14)	
	RF	0.90 (0.90-0.91)		0.92 (0.91-0.93)		0.89 (0.88-0.89)		0.639	8.23 (7.97-8.49)		0.09 (0.08-0.10)	
**10%**												
	MEWS	0.70 (0.69-0.71)		0.72 (0.70-0.73)		0.69 (0.68-0.69)		0.419	2.29 (2.23-2.35)		0.41 (0.39-0.43)	
	LR	0.87 (0.86-0.87)		0.86 (0.85-0.87)		0.87 (0.87-0.88)		0.675	6.80 (6.54-7.07)		0.16 (0.15-0.17)	
	RNN	0.89 (0.89-0.90)		0.93 (0.92-0.94)		0.85 (0.85-0.86)		0.681	6.32 (6.11-6.54)		0.08 (0.07-0.09)	
	RF	0.92 (0.92-0.92)		0.94 (0.94-0.95)		0.90 (0.89-0.90)		0.756	9.31 (8.95-9.69)		0.06 (0.06-0.07)	

^a^BA: balanced accuracy.

^b^PLR: positive likelihood ratio.

^c^NLR: negative likelihood ratio.

^d^MEWS: modified early warning score.

^e^LR: logistic regression.

^f^RNN: recurrent neural network.

^g^RF: random forest.

The RF models showed the best overall predictive performance in the validation cohort for each balancing ratio. For instance, the AUROC of RF was 0.97 and an AUPRC of 0.86, while RNN, LR, and MEWS had AUROCs of 0.95, 0.92, and 0.76 and AUPRCs of 0.82, 0.72, and 0.42, respectively, in the validation cohort with 10% balancing. The RF-based models outperformed the RNN- and LR-based models as well as MEWS in terms of all performance measures in class prediction (all *P*<.001). The RF-based models showed better overall prediction performance, and all performance measures for the class prediction improved as higher balancing ratios were applied for the class imbalance adjustment (eg, the AUPRC and F1 score improved from 0.37 to 0.86 and from 0.20 to 0.76, respectively).

After considering all the factors, the RF-based model with a 10% balancing ratio was selected as the best model. The best model had a sensitivity and specificity of 0.94 (95% CI 0.94-0.95) and 0.90 (95% CI 0.89-0.90), respectively. Moreover, the positive likelihood ratio value of 9.31 (95% CI 8.95-9.69) and the negative likelihood ratio value of 0.06 (95% CI 0.06-0.07) indicate that the model is clinically informative and very useful in practice. For the best model, the ICI and calibration slope were 0.01 and 1.14 (95% CI 1.09-1.18), respectively. Table 5 summarizes the importance of each predictor in the best model. Body temperature and SpO_2_ were relatively important predictors.

**Table 5:** Predictor importance in the random forest model with 10% balancing.

Feature	Predictor importance, mean (SD)
Body temperature	0. 284 (0.014)
Peripheral oxygen saturation	0.232 (0.011)
Heart rate	0.127 (0.005)
Duration	0.096 (0.009)
Respiratory rate	0.084 (0.005)
Systolic blood pressure	0.081 (0.006)
Diastolic blood pressure	0.072 (0.005)
Chief concern	0.012 (N/A)^a^
Age	0.010 (N/A)
Sex	0.002 (N/A)

^a^N/A: not applicable.

## Discussion

### Principal Findings

Recent prediction model guidelines emphasize validation and clinical application [6,24]. Clinical usage of prediction models is important; therefore, these models should be clinically adaptable and persuasive. However, previous studies are lacking in these aspects. In the present study, we attempted to remedy this by verifying the suitability of the model using chronological visualization focused on clinical usability.

In this study, we developed the model using the method of generating sequential data vectors. The comprehensive model validation considered performance and various clinical relevance aspects. The clinical validity of the model was assessed through visualization of chronological characteristics.

Machine learning–based prediction models are often called “black boxes” because the algorithms provide answers without any “human” knowledge. When calculations and suggestions cannot be clinically explained, it is almost impossible to apply them in real-world settings. One reason for this is that it is not clear who or what is responsible for clinical decisions [25,26]. Another reason is that clinicians are not notified of the parameters on which they should focus; thus, applying machine learning–based prediction in a clinical setting may be confusing.

It can be practically important to suggest a single unified threshold for class prediction across all prediction time points. The best threshold chosen with the highest balanced accuracy at each prediction time point ranged from 0.30 to 0.40. Within this range, we considered several candidates for the unified threshold and investigated their performance in various aspects (Multimedia Appendix 4-6). A unified threshold of 0.35 was selected because of its stable performance and considerable net benefit. Clinicians can apply either a single unified threshold across all time points or the best threshold for each time point based on practicality and depending on their environment.

In practice, clinicians can apply the proposed prediction process as follows. When a new patient visits the ED, the initial assessment is conducted and the initially assessed predictors are recorded. Then, the patient’s vital signs are monitored and the sequential measurements are converted into a sequential record for serially assessed predictors. Then, the developed prediction model produces the predicted probability of cardiac arrest. When a vital sign is updated, the sequential record is promptly updated and used as a new input to update the predicted probability. Based on a prechosen threshold (eg, 0.35), the risk of the patient at the moment is classified as high if the prediction probability is greater than or equal to the threshold. This prediction process can be applied as a trigger alarm system, in which the high-risk prediction initiates more intensive care or closer monitoring. In this case, the missed rate and the false alarm rate are expected to be 6% and 10%, respectively. Therefore, the increase in the workload of the medical team is only 10%.

Significant efforts have been made to improve the explainability of prediction models so they can be applied in real-world settings [27-30]. Due to the nature of machine learning, it is difficult to explain individual decisions specifically; however, it is still possible to describe the overall decision process based on feature importance [31,32]. On average, body temperature and SpO_2_ were important, especially in the 1st, 2nd, 3rd, and 10th measurements. The remaining features were relatively important only in the 10th measurement. Our findings demonstrate that there is a difference in the importance of these features over time. Thus, it is necessary to test the performance by narrowing the time interval.

Another factor that affects the clinical validity of prediction models is imbalance of the outcome parameters [33,34]. It is clinically valuable to know how performance changes in different settings, and this change was given little attention in previous studies. In this study, we used various structures and considered both model accuracy and realistic settings to demonstrate the statistical robustness of the model.

In the real world, serially assessed vital data often contain missing values for various reasons, and these data should be handled properly and efficiently. When choosing missing-handling methods, we focused on two factors: the nonrandomness of missing patterns in the ED data and the applicability to the risk prediction of a new patient in a real ED situation. In the process of sequential data set generation, nonrandom missing data naturally occurs due to the lack of information on vital signs at early prediction time points (ie, before assessing vital signs 10 times). We attempted to use this nonrandom missing pattern as additional information by zero imputation, which may reflect the low frequency of vital assessment to a certain degree. Moreover, at other prediction time points, missing values occur nonrandomly because vital assessments in the ED are ordered according to the patient’s condition and are also monitored periodically. We suggested filling in the missing data with the most recent value because it is practically applicable to the prediction of risk for new patients based on our prediction procedure.

### Limitations

Our study has several limitations. First, the use of machine learning algorithms was limited, and the study design (eg, risk period and number of sequential measurements) was set heuristically based on clinical experience in real clinical settings. However, a prediction model can be developed by applying the process in this study using other algorithms as well. Second, because this study was conducted in a single department of a single center, it is not representative. To use the model in other institutions, further external validation should be performed. Third, few features were used, and the results of other tests containing significant information (eg, laboratory tests) were not considered. Using this additional information may be advantageous, although the proposed model is already considerably accurate. Finally, the outcome was an ultimate result (ie, cardiac arrest) and did not include resuscitation efforts or prescription. In a clinical setting, resuscitation efforts should be considered. Therefore, it is necessary to extend the proposed method to include resuscitation and acute deterioration.

### Conclusions

In this study, we developed a cardiac arrest prediction model in the ED using machine learning and sequential characteristics. The model was validated for clinical usefulness using chronological visualization focused on clinical usability.

## Appendix

Multimedia Appendix 1 Patient characteristics after propensity score matching.

Multimedia Appendix 2 Supplemental code for developing the models.

Multimedia Appendix 3 ROC and calibration curves in the development and validation cohorts.

Multimedia Appendix 4 Time-point performance in class prediction of the candidate threshold systems.

Multimedia Appendix 5 Overall performance in class prediction of the candidate threshold systems.

Multimedia Appendix 6 Decision curve for the best prediction model.

## Abbreviations

AUPRC: area under the precision recall curve
AUROC: area under the receiver operating characteristic curve
BA: balanced accuracy
CDSS: clinical decision support system
ED: emergency department
ICI: integrated calibration index
LR: logistic regression
MEWS: modified early warning score
NEDIS: National Emergency Department Information System
RF: random forest
RNN: recurrent neural network
SpO_2_: peripheral oxygen saturation
