# Supplementary material for: Prediction of Cardiac Arrest in the Emergency Department Based on Machine Learning and Sequential Characteristics: Model Development and Retrospective Clinical Validation Study
Source: JMIR Med Inform. 2020 Aug 4;8(8):e15932. doi: 10.2196/15932 (PMC7435618; doi:10.2196/15932)
Supplement: Multimedia Appendix 1 [file medinform_v8i8e15932_app1.pdf]

Multimedia Appendix 1. Patient characteristics after propensity score matching.

The following results obtained from R matchit function with the default setting (i.e., nearest method and logit distance). The standardized mean difference (SMD) is used to compare patient characteristics between development and validation cohorts.

Table A1. Patient characteristics for development and validation cohorts with 1% balancing.

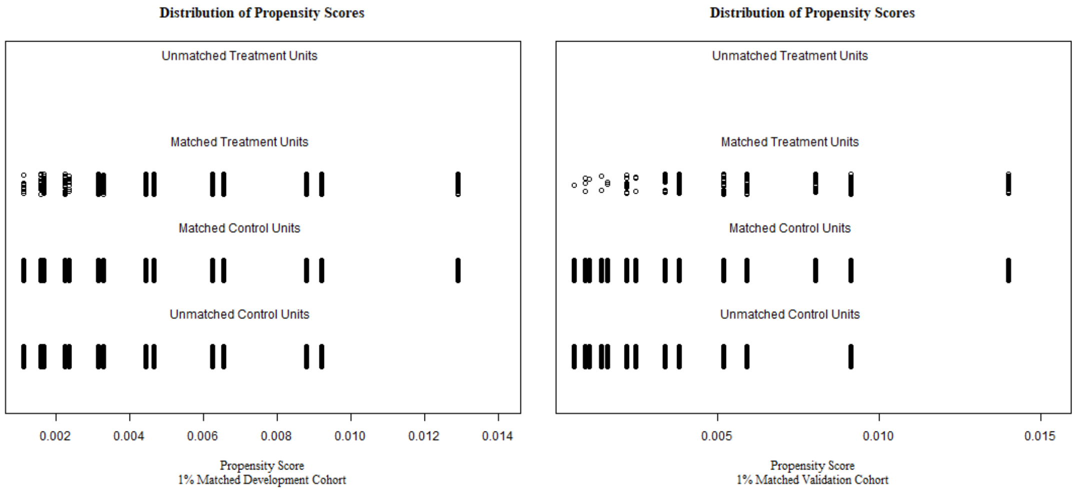

	**Development cohort**			**Validation cohort**			**SMD**
**Characteristics**	**Event (n = 791)**	**Non-event (n = 78,309)**	***P* Value**	**Event (n = 202)**	**Non-event (n = 19,998)**	***P* Value**	
Demographic							
Sex, n (%)			1.000			0.859	0.103
Male	472 (59.7)	46,728 (59.7)		133 (65.8)	13,335 (66.7)		
Female	319 (40.3)	31,581(40.3)		69 (34.2)	6,663 (33.3)		
Age, mean (SD), y	65.2 (15.6)	64.8 (15.2)	0.463	68.3 (13.6)	68.1 (13.3)	0.807	0.215
Vital signs, mean (SD)							
Blood pressure, mm Hg							
Systolic	112.6 (25.5)	122.1 (25.2)	< .001	112.9 (28.4)	123.0 (25.8)	< .001	0.039
Diastolic	65.0 (15.9)	72.1 (15.1)	< .001	64.3 (16.4)	72.1 (15.1)	< .001	0.001
BT, °C	36.7 (2.4)	36.9 (1.8)	< .001	36.8 (2.1)	37.0 (2.2)	< .001	0.040
HR, beats/min	99.9 (23.7)	88.3 (21.1)	< .001	99.0 (22.1)	87.9 (20.9)	< .001	0.033
RR, breaths/min	21.2 (6.6)	19.9 (4.1)	< .001	20.6 (6.4)	19.2 (3.9)	< .001	0.153
SpO_2, %	94.9 (11.0)	91.8 (22.2)	< .001	95.2 (8.7)	96.7 (7.5)	< .001	0.270

Table A2. Patient characteristics for development and validation cohorts with 5% balancing.

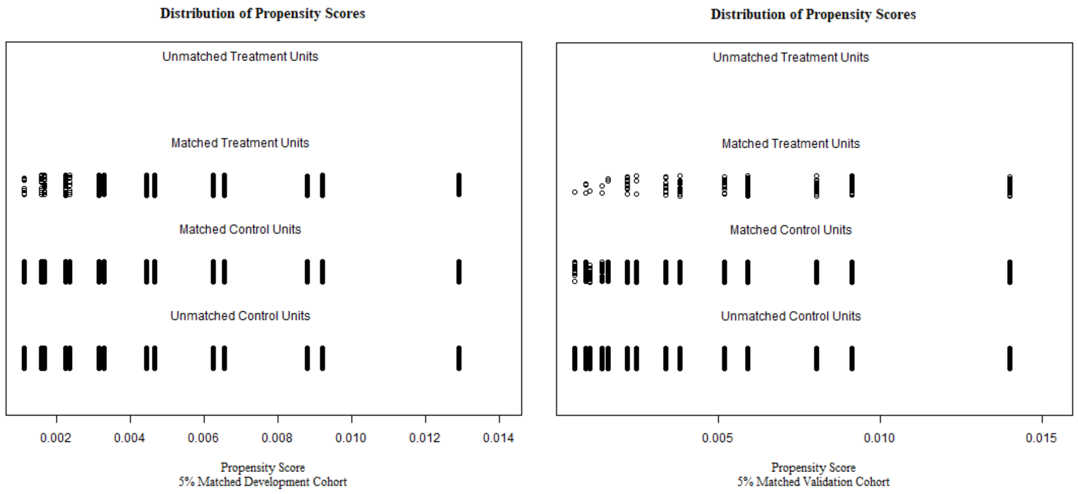

	Development cohort			Validation cohort			SMD
	Event	Non-event		Event	Non-event		
**Characteristics**	**(n = 791)**	**(n = 15,029)**	***P* Value**	**(n = 202)**	**(n = 3,838)**	***P* Value**	
Demographic							
Sex, n (%)			1.000			1.000	0.039
Male	472 (59.7)	8,968 (59.7)		133 (65.8)	2,527 (65.8)		
Female	319 (40.3)	6,061(40.3)		69 (34.2)	1,311(34.2)		
Age, mean (SD), y	65.2 (15.6)	64.8 (15.3)	0.566	68.3 (13.6)	68.5 (13.8)	0.837	0.251
Vital signs, mean (SD)							
Blood pressure, mm Hg							
Systolic	112.6 (25.5)	122.2 (25.1)	< .001	112.9 (28.4)	123.0 (26.2)	< .001	0.041
Diastolic	65.0 (15.9)	72.1 (15.0)	< .001	64.3 (16.4)	71.8 (15.6)	< .001	0.008
BT, °C	36.7 (2.4)	36.9 (1.9)	< .001	36.8 (2.1)	36.9 (2.8)	< .001	0.017
HR, beats/min	99.9 (23.7)	88.2 (20.8)	< .001	99.0 (22.1)	87.7 (20.9)	< .001	0.055
RR, breaths/min	21.2 (6.6)	19.9 (4.0)	< .001	20.6 (6.4)	19.2 (3.9)	< .001	0.136
SpO_2, %	94.9 (11.0)	91.5 (22.9)	< .001	95.2 (8.7)	96.5 (8.2)	< .001	0.207

Table A3. Patient characteristics for development and validation cohorts with 10% balancing.

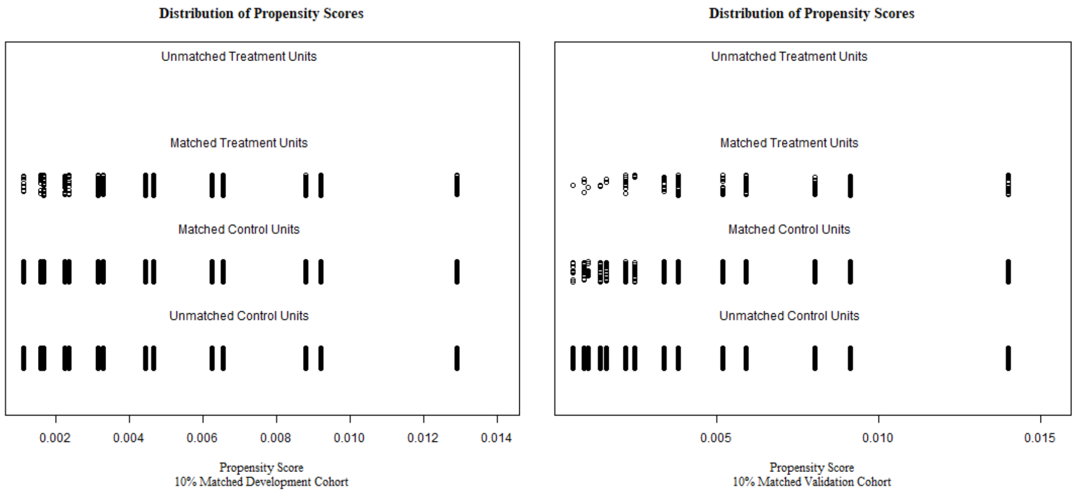

Characteristics	**Development cohort**			**Validation cohort**			**SMD**
	**Event (n = 791)**	**Non-event (n = 7,119)**	***P* Value**	**Event (n = 202)**	**Non-event (n = 1,818)**	***P* Value**	
Demographic							
Sex, n (%)			1.000			1.000	0.001
Male	472 (59.7)	4,248 (59.7)		133 (65.8)	1,197 (65.8)		
Female	319 (40.3)	2,871 (40.3)		69 (34.2)	621 (34.2)		
Age, mean (SD), y	65.2 (15.6)	64.9 (15.3)	0.637	68.3 (13.6)	68.4 (13.7)	0.916	0.264
Vital signs, mean (SD)							
Blood pressure, mm Hg							
Systolic	112.6 (25.5)	122.4 (25.4)	< .001	112.9 (28.4)	123.1 (25.2)	< .001	0.026
Diastolic	65.0 (15.9)	72.0(15.1)	< .001	64.3 (16.4)	72.2 (14.9)	< .001	0.013
BT, °C	36.7 (2.4)	36.9 (1.9)	< .001	36.8 (2.1)	36.9 (2.7)	< .001	0.011
HR, beats/min	99.9 (23.7)	88.8 (20.8)	< .001	99.0 (22.1)	87.6 (21.4)	< .001	0.056
RR, breaths/min	21.2 (6.6)	20.0 (4.1)	< .001	20.6 (6.4)	19.3 (4.0)	< .001	0.107
SpO_2, %	94.9 (11.0)	91.9 (21.9)	< .001	95.2 (8.7)	96.4 (8.2)	< .001	0.148
