# Supplementary material for: Prediction of Cardiac Arrest in the Emergency Department Based on Machine Learning and Sequential Characteristics: Model Development and Retrospective Clinical Validation Study
Source: JMIR Med Inform. 2020 Aug 4;8(8):e15932. doi: 10.2196/15932 (PMC7435618; doi:10.2196/15932)
Supplement: Multimedia Appendix 2 [file medinform_v8i8e15932_app2.pdf]

Multimedia Appendix 2. Supplemental Code for Developing Models

[ ]:
```
# ================================================
# The version of library
# - scikit-learn (0.21.3)
# - keras (2.3.1)
# ================================================

# Call library
import pandas as pd
import numpy as np
import random as rn

# Cross Validation & repeated stratified K-Fold & GridSearch
from sklearn.model_selection import RepeatedStratifiedKFold
from sklearn.model_selection import GridSearchCV

# Model Classifier
from sklearn.linear_model import LogisticRegression
from sklearn.ensemble import RandomForestClassifier
import keras
from keras.utils.np_utils import to_categorical
from keras.models import Sequential, load_model
from keras.layers import Activation, Dense, Dropout, LSTM
from keras import optimizers
from keras.wrappers.scikit_learn import KerasClassifier
```

[ ]:
```
### You can change the settings(e.g., hyperparameter candidates) to suit␣
↪environment
```

[ ]:
```
# Stratified Cross Validation : 10-fold 5-repeated
rskfold = RepeatedStratifiedKFold(n_splits=10, n_repeats=5, random_state=2864)
```

1 Logistic Regression : Ridge

[ ]:
```
# Define model : Logistic Regression-Ridge penalty
LR_model = LogisticRegression(solver='liblinear', penalty='l2',
                              multi_class='ovr', random_state=2864)
```

```
# Hyperparameter Optimization
param_grid = {'C': [0.001, 0.01, 0.1, 1, 10, 100, 1000]}
LR_M = GridSearchCV(LR_model, param_grid, cv=rskfold,
                    return_train_score=True, scoring='accuracy')

# Start model fitting
LR_M.fit(train_x, train_y)

# Check the performance
LR_M.score(test_x, test_y)
```

[ ]:

```

```

2 Random Forest

[ ]:

```
# Define model : Random Forest
RF_model = RandomForestClassifier(criterion='entropy', random_state=2864)

# Hyperparameter Optimization
param_grid = [{'max_features': ['auto', 'sqrt', 'log2'],
               'max_depth' : [10, 30, None]}]
RF_M = GridSearchCV(RF_model, param_grid, cv=rskfold,
                    return_train_score=True, scoring='accuracy')

# Start model fitting
RF_M.fit(train_x, train_y)

# Check the performance
RF_M.score(test_x, test_y)
```

[ ]:

```

```

3 Recurrent Neural Network - LSTM

[ ]:

```
# Change the dataset input shape for deep learning
X_train = np.array(x_train);Y_train = np.array(y_train)
X_test = np.array(x_test);Y_test = np.array(y_test)
X_train = X_train.reshape(X_train.shape[0], 1, X_train.shape[1])
X_test = X_test.reshape(X_test.shape[0], 1, X_test.shape[1])
# Input dimension
input_dim = X_train.shape[2]
```

[ ]:

```
# Define model
def create_model(optimizer='adam'):
```

```
    # Create model
    model = Sequential()
    model.add(LSTM(32, input_shape=(1,input_dim), return_sequences=True))
    model.add(LSTM(32, return_sequences=True))
    model.add(LSTM(16))
    model.add(Dense(1, activation='sigmoid'))
    # Compile model
    model.compile(loss='binary_crossentropy',
                  optimizer = optimizer, metrics=['accuracy'])
    return model

RNN_model = KerasClassifier(build_fn=create_model, epochs=10, batch_size=128,
                            verbose=0, shuffle=True)
```

[ ]:
```
# Hyperparameter Optimization
param_grid_rnn = {'optimizer' : ['SGD', 'RMSprop', 'Adam']}
RNN_M = GridSearchCV(RNN_model, param_grid_rnn, cv=rskfold,
                     return_train_score=True, scoring='accuracy')

# Start model fitting
RNN_M.fit(X_train, Y_train)

# Check the performance
RNN_M.score(X_test, Y_test)
```

[ ]:
```

```

[ ]:
```
# Next steps evaluate common performance
# e.g.) confusion matrix, best cutoff explore,
# discrimination, calibration, decision curve analysis, etc...
```
