# Supplementary material for: Prediction of Cardiac Arrest in the Emergency Department Based on Machine Learning and Sequential Characteristics: Model Development and Retrospective Clinical Validation Study
Source: JMIR Med Inform. 2020 Aug 4;8(8):e15932. doi: 10.2196/15932 (PMC7435618; doi:10.2196/15932)

Multimedia Appendix 3. ROC and calibration curves in development and validation cohorts.

Figure A1. ROC curves for models in development and validation cohorts with 10% balancing.

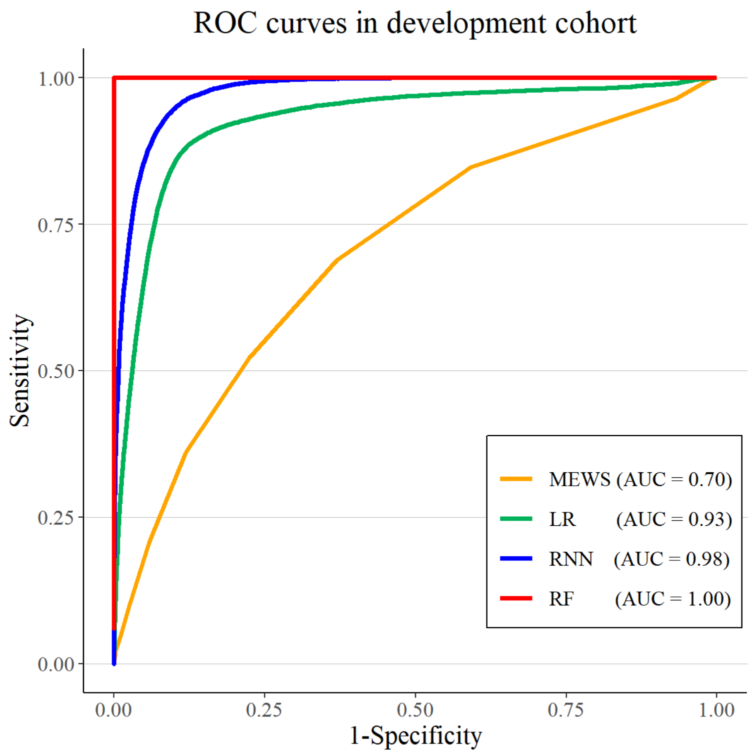

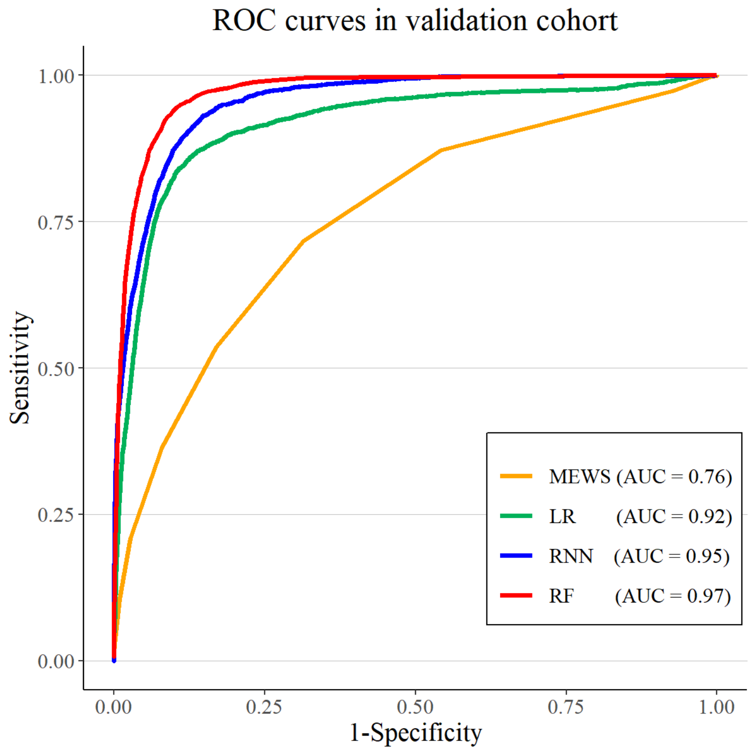

Figure A2. Calibration curves for models in validation cohort. The x-axis represents predicted outcome probability. The y-axis represents observed outcome probability.

Calibration curves for models in validation cohort with 10% balancing

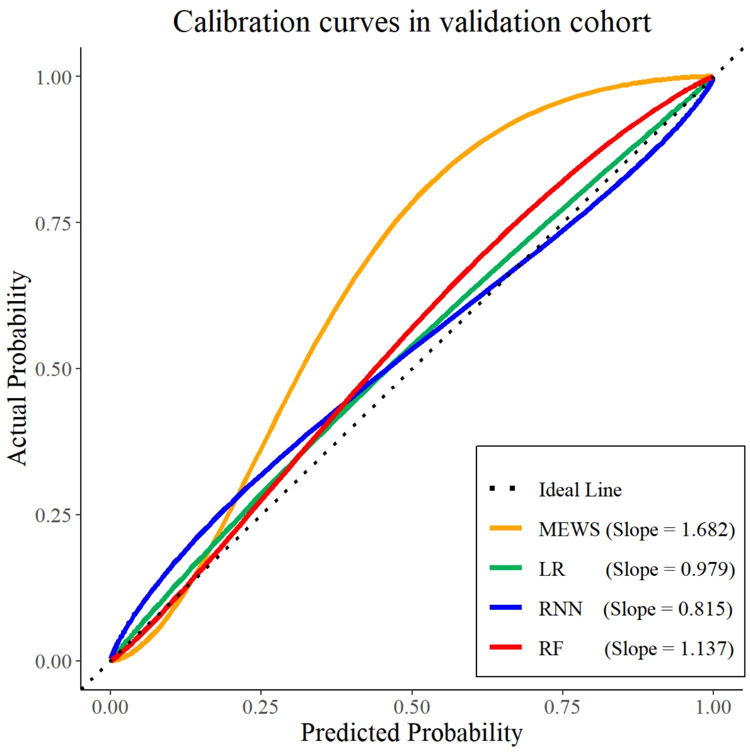

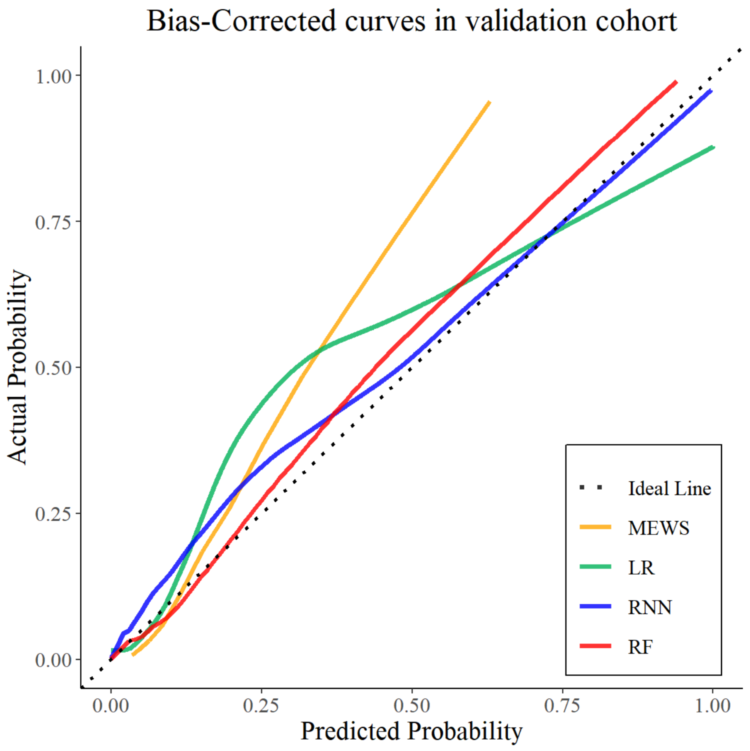

Calibration curves for models in validation cohort with imbalance adjustment

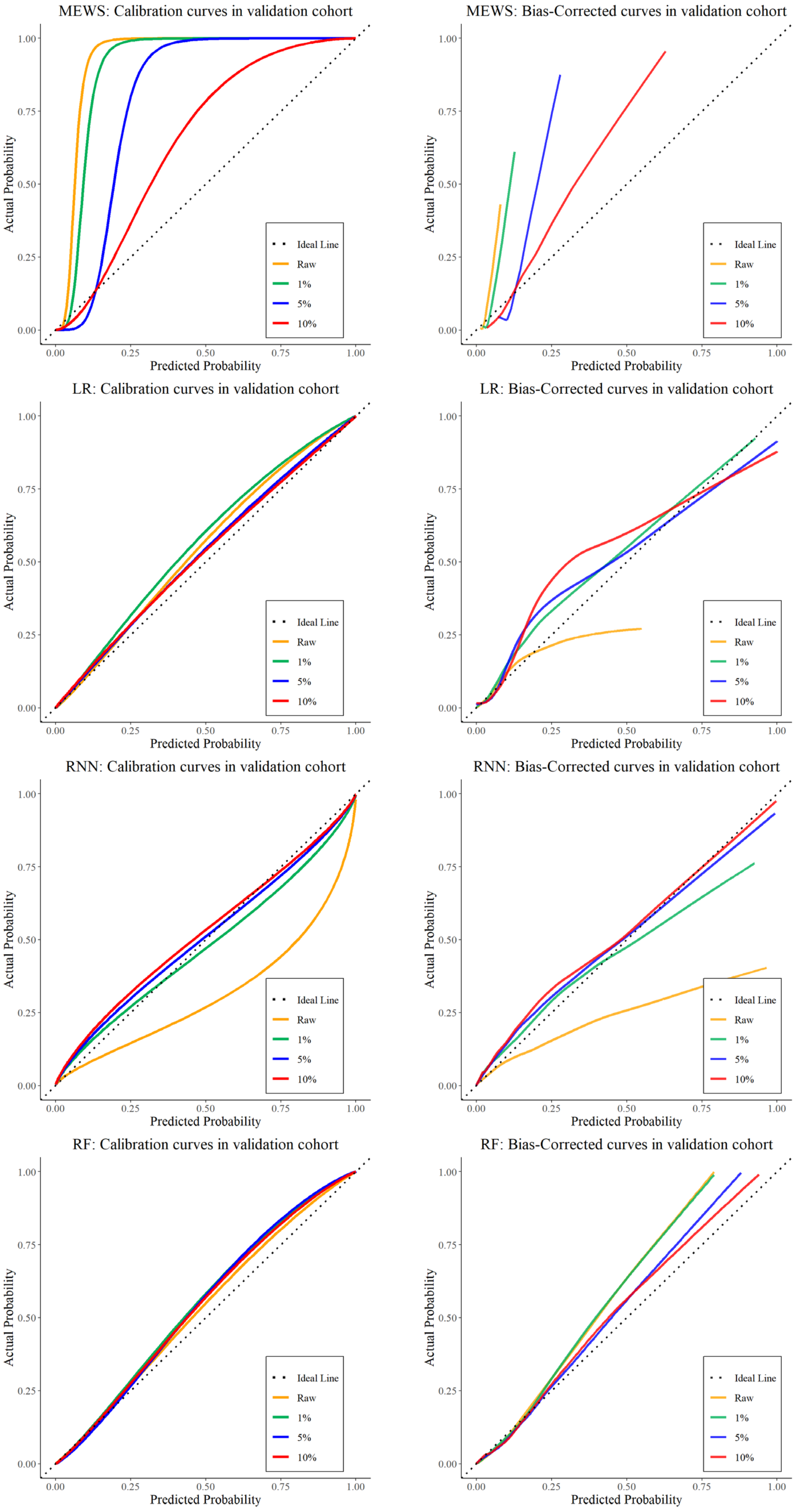

Supplement: Multimedia Appendix 3 [file medinform_v8i8e15932_app3.pdf]
