# Supplementary material for: Prediction of Cardiac Arrest in the Emergency Department Based on Machine Learning and Sequential Characteristics: Model Development and Retrospective Clinical Validation Study
Source: JMIR Med Inform. 2020 Aug 4;8(8):e15932. doi: 10.2196/15932 (PMC7435618; doi:10.2196/15932)

**Multimedia Appendix 4.** Time-point performance in class prediction of candidate threshold systems. The x-axis represents prediction time point (h) before event occurrence. The y-axis represents balanced accuracy. Cut:Best indicates the candidate threshold system that uses the best threshold with the highest balanced accuracy at each prediction time-point. Cut:0.30, Cut:0.35 and Cut:0.40 indicate threshold systems, each of which uses a unified threshold over all prediction time-points.

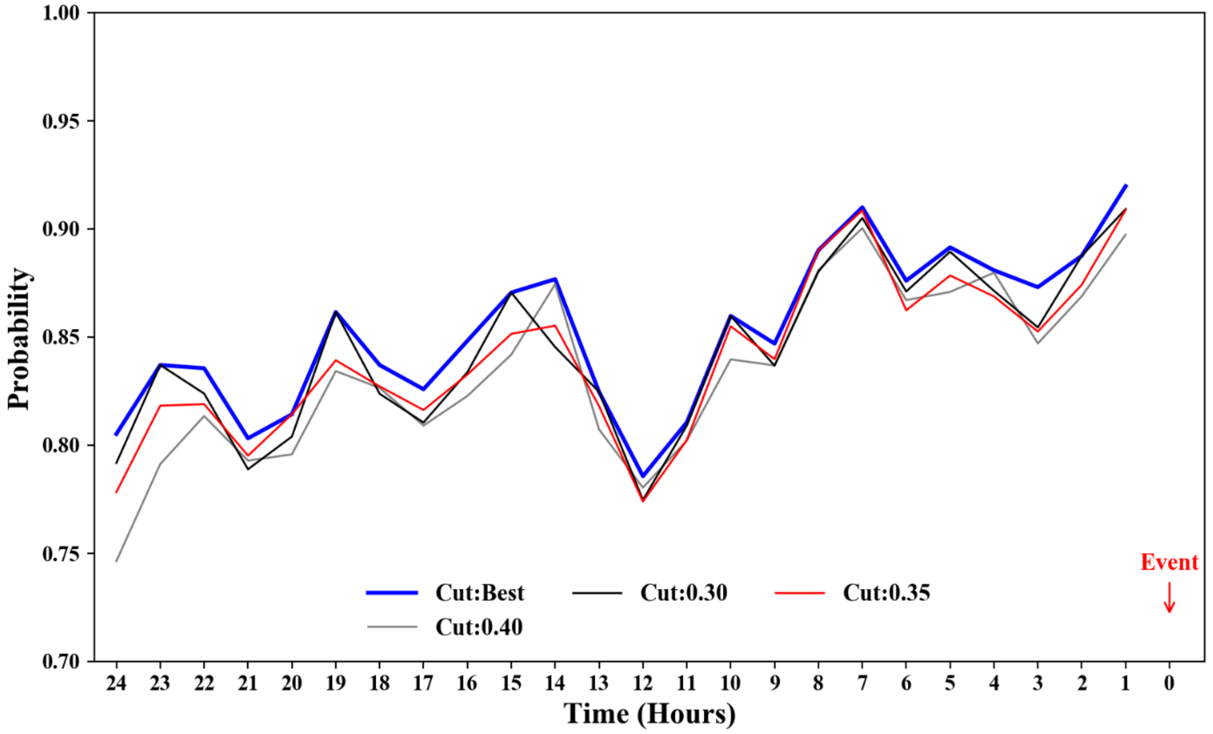

Supplement: Multimedia Appendix 4 [file medinform_v8i8e15932_app4.pdf]
