# Supplementary material for: Prediction of Cardiac Arrest in the Emergency Department Based on Machine Learning and Sequential Characteristics: Model Development and Retrospective Clinical Validation Study
Source: JMIR Med Inform. 2020 Aug 4;8(8):e15932. doi: 10.2196/15932 (PMC7435618; doi:10.2196/15932)
Supplement: Multimedia Appendix 5 [file medinform_v8i8e15932_app5.pdf]

Multimedia Appendix 5. Overall performance in class prediction of candidate threshold systems.

Statistics	Threshold	Balanced accuracy	F1	NLR	PLR	Precision	Sensitivity	Specificity
Mean	0.30	0.8444	0.7976	0.1659	5.3359	0.7431	0.8641	0.8247
	0.35	0.8409	0.7946	0.2053	6.1786	0.7704	0.8247	0.8571
	0.40	0.8345	0.7884	0.2395	6.9894	0.7910	0.7903	0.8787
	Best	0.8530	0.8070	0.1577	6.0200	0.7608	0.8672	0.8389
SD	0.30	0.0373	0.0361	0.0645	1.8597	0.0478	0.0510	0.0439
	0.35	0.0368	0.0356	0.0706	1.9731	0.0477	0.0598	0.0343
	0.40	0.0400	0.0402	0.0783	2.2329	0.0439	0.0677	0.0298
	Best	0.0358	0.0378	0.0716	2.1910	0.0631	0.0631	0.0542
