# Supplementary material for: Prediction of Cardiac Arrest in the Emergency Department Based on Machine Learning and Sequential Characteristics: Model Development and Retrospective Clinical Validation Study
Source: JMIR Med Inform. 2020 Aug 4;8(8):e15932. doi: 10.2196/15932 (PMC7435618; doi:10.2196/15932)

Multimedia Appendix 6. Decision curve for the best prediction model. The x-axis represents threshold for prediction probability. The y-axis represents net benefit of prediction model based on threshold.

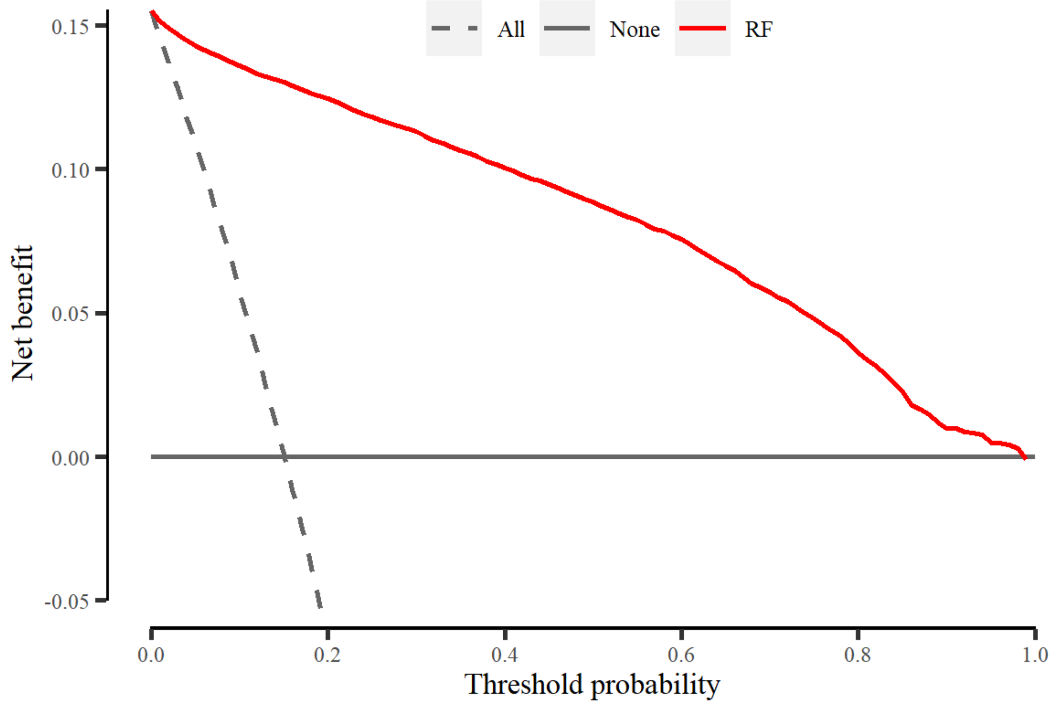

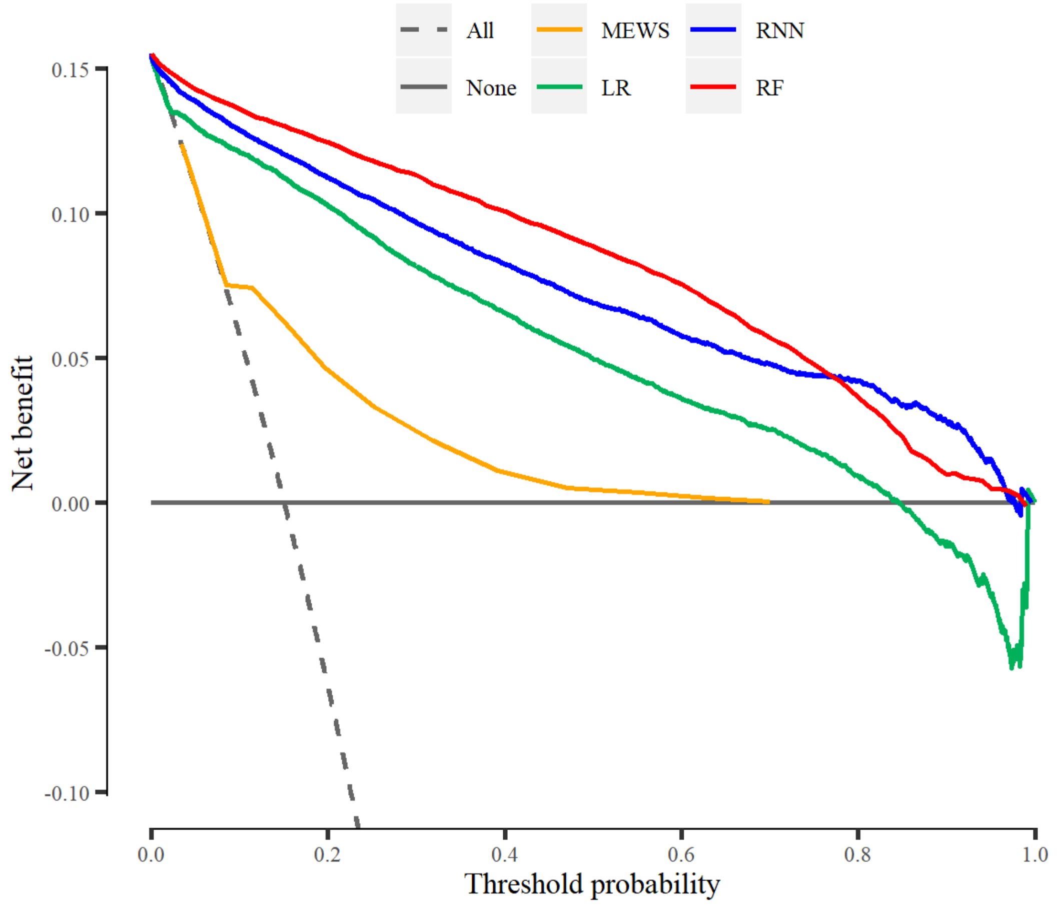

Supplement: Multimedia Appendix 6 [file medinform_v8i8e15932_app6.pdf]
